# Apple Derived Cellulose Scaffolds for 3D Mammalian Cell Culture

**DOI:** 10.1371/journal.pone.0097835

**Published:** 2014-05-19

**Authors:** Daniel J. Modulevsky, Cory Lefebvre, Kristina Haase, Zeinab Al-Rekabi, Andrew E. Pelling

**Affiliations:** 1 Centre for Interdisciplinary NanoPhysics, University of Ottawa, Ottawa, Ontario, Canada; 2 Department of Biology, University of Ottawa, Ottawa, Ontario, Canada; 3 Department of Physics, University of Ottawa, Ottawa, Ontario, Canada; 4 Institute for Science, Society and Policy, University of Ottawa, Ottawa, Ontario, Canada; Instituto Butantan, Brazil

## Abstract

There are numerous approaches for producing natural and synthetic 3D scaffolds that support the proliferation of mammalian cells. 3D scaffolds better represent the natural cellular microenvironment and have many potential applications *in vitro* and *in vivo*. Here, we demonstrate that 3D cellulose scaffolds produced by decellularizing apple hypanthium tissue can be employed for *in vitro* 3D culture of NIH3T3 fibroblasts, mouse C2C12 muscle myoblasts and human HeLa epithelial cells. We show that these cells can adhere, invade and proliferate in the cellulose scaffolds. In addition, biochemical functionalization or chemical cross-linking can be employed to control the surface biochemistry and/or mechanical properties of the scaffold. The cells retain high viability even after 12 continuous weeks of culture and can achieve cell densities comparable with other natural and synthetic scaffold materials. Apple derived cellulose scaffolds are easily produced, inexpensive and originate from a renewable source. Taken together, these results demonstrate that naturally derived cellulose scaffolds offer a complementary approach to existing techniques for the *in vitro* culture of mammalian cells in a 3D environment.

## Introduction

Development of novel biomaterials for the *in vitro* culture of cells in three-dimensional (3D) microenvironments has gained traction in recent years [1]–[6]. The motivation behind this development is to compensate for limitations of current two-dimensional (2D) cell culture practices. In particular, 2D plastic or glass substrates are ubiquitously employed to study many biological processes, despite the obvious structural and mechanical differences with the *in vivo* microenvironment. *In vivo*, cells are found in a complex extracellular matrix (ECM) whose biochemical and physical properties have a significant impact on numerous critical physiological and pathological processes [7]. Significant morphological and biological differences have already been observed between cells grown on 2D versus 3D microenvironments [8], [9]. It is has been routinely observed that primary cells isolated from tissues will become progressively flatter when cultured on conventional 2D surfaces [10], [11]. Conversely, cells cultured on 2D surfaces can regain their 3D morphologies when placed into a 3D culture scaffold [12]. 3D cell culture promises to more closely reflect the biochemical and physical properties of the cellular microenvironment found in tissues and organs [13], and so the development of novel biomaterials towards this effort is of considerable importance.

Both synthetic and naturally derived materials are currently employed in 3D culture methods, in order to create tuneable scaffolds engineered with specific biochemical and physical properties. Cellulose, the major component of plant cell walls, is an organic polysaccharide made of D-glucose subunits through β(1–4) bonds. Unlike the polysaccharides starch and glycogen, cellulose provides very little nutritional energy as the β(1–4) glycosidic bonds are difficult to digest and can only be broken down by cellulase [14]. As such, there has a great focus on using cellulose as a candidate biomaterial [12], [15]–[23]. Cellulose has previously been employed as a permeable dialysis membrane and as diffusion limiting membranes within biosensors[24]. As well, previous studies found that cellulose produced by bacteria could support the proliferation of mammalian cells [20], [25], [26]. Synthetically produced cellulose scaffolds have also been employed for 3D mammalian cell culture [2], [19], [21], [23]. Myocytes cultured on these synthetic cellulose scaffolds contained periodic myofibrils, a distinct cytoarchitectural element within mature cardiac myocytes [27]. As well, enhanced connectivity, in the form of increased gap junction density, and electrochemical connectivity, resulted from 3D culture, in comparison to cells grown on glass [27]. These examples suggest that cellulose may be a suitable material to support 3D cell growth. Moreover, cellulose is widely available as it is the most common organic polymer, accounting for 1.5×10^12^ tons of total annual biomass production [28].

Apple hypanthium tissue has an internal structure composed of cell walls that encompass pores and air pockets, facilitating the transport of nutrients and water throughout the fleshy tissue. These naturally developed characteristics, are important in any scaffold employed for 3D cell culture [29]. In order to act as a 3D scaffold, the apple hypanthium tissue must first be decellularized in order to remove existing nucleic acids, lipids, and proteins, producing a purified cellulose scaffold. Decellularization is now commonly employed on mammalian tissues to selectively remove cellular components while leaving behind an intact ECM [30]–[33] Typically, mammalian tissues are bathed in solutions, detergents and/or proteases, in order to produce a decellularized matrix that retains the shape of the original tissue[34]–[43]. Decellularized tissue can then be repopulated with new cells, in order to produce new functional organs. Hearts, kidneys have been decellularized and reseeded with various cells [33], [41]–[43]. As well, functional bladders and lungs have be produced and transplanted into animals using this technique [44], [45]. Importantly, decellularized tissue also maintains a well conserved native ECM architecture and cell-ECM binding domains [41].

In this study, we hypothesized that decellularized apple hypanthium tissue might provide an easily produced scaffold for 3D cell culture. The major aim of this study was to demonstrate that mammalian cells would successfully proliferate within a 3D cellulose scaffold in vitro. Through modification of an existing decellularization protocol, we generated apple-derived cellulose scaffolds for cell culture. We examined how three mammalian cell types (mouse NIH3T3 fibroblasts, mouse C2C12 myoblasts and human HeLa epithelial cells) proliferated within these scaffolds, for up to twelve weeks. Phase contrast microscopy, laser scanning confocal microscopy and scanning electron microscopy were used to characterize the structure of the scaffolds, cell growth, cell morphology and the influence of the scaffolds on the actin cytoskeleton. We also modified the surface biochemistry and mechanical properties of the cellulose scaffolds by collagen functionalization, or chemical cross-linking with glutaraldehyde. Atomic force microscopy was employed to quantify the effect of these modifications on the mechanical properties of the scaffolds. We demonstrate that the 3 mammalian cell lines used in this study were able to proliferate and remain viable in the 3D cellulose scaffold in vitro, achieving cell densities similar to other synthetic and natural biomaterials. Given the natural porosity and ease of production of cellulose scaffolds, as well as the ability to modify their mechanical properties, we demonstrate that cellulose scaffolds are a potentially useful biomaterial that can be successfully employed for in vitro 3D cell culture.

## Materials and Methods

### Apple tissue preparation, decellularization and storage

**Figure 1:**
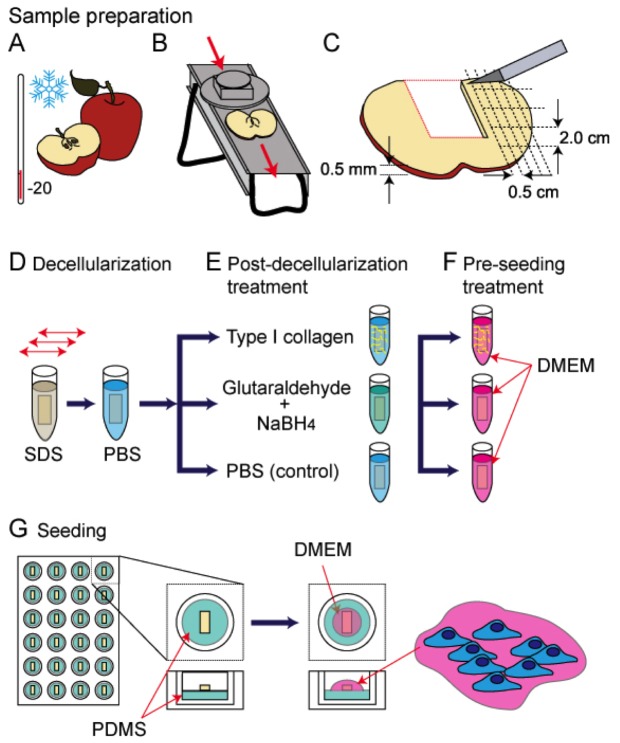
A cartoon schematic representing the apple tissue decellularization and mammalian cell seeding protocol used in this study. A) McIntosh Red apples were exposed to −20°C temperatures for a max duration of 5 minutes, to increase the firmness of the outer apple hypanthium tissue. B) Uniform 1.2±0.1 mm thick slices of the apples were obtained using a mandolin slicer. Slices containing any of the ovary core of the apple were removed. C) The apple slices were cut into uniform 2.0 by 0.5 cm segments that were placed in individual microcentrifuge tubes. D) A 0.5% SDS solution was added to the microcentrifuge tubes and placed on a shaker for 12 hours at room temperature. The scaffolds were then rinsed repeatedly with PBS and allowed to incubate in a PBS solution with 1% streptomycin/penicillin and 1% amphotericin B for 6 hours at room temperature. E) The scaffolds were then coated with Type 1 collagen, chemically cross linked with glutaraldehyde or incubated in PBS. F) All the samples were then incubated in mammalian cell culture medium (DMEM) for 12 hours in a standard tissue culture incubator maintained at 37°C and 5% CO_2_. G) The scaffolds were placed in PDMS coated 24 well plates and a 40 µL cell suspension was placed on each. After 6 hours in the incubator the wells were filled with DMEM and cells cultured for up to 12 weeks.

McIntosh Red apples (Canada Fancy) were stored at 4°C in the dark for a maximum of two weeks. In order to prepare apple sections, the fruit was first chilled in a −20°C freezer for 5 minutes prior to being cut with a mandolin slicer to a uniform thickness of 1.20±0.14 mm, measured with a vernier caliper (Fig. 1 A-B). Only the outer (hypanthium) tissue of the apple was used. Slices containing visible ovary-core tissue were not used. The slices were then cut into 2.0×0.5 cm segments parallel to the direction of the apple pedicel (Fig. 1 C). Apple tissue was then decellularized by using a well-established protocol [41] for removing cellular material and DNA from tissue samples while leaving behind an intact and three-dimensional scaffold. Individual apple tissue samples were placed in sterilized 2.5 mL microcentrifuge tubes and 2 mL of 0.5% sodium dodecyl sulphate (SDS) (Sigma-Aldrich) solution was added to each tube. Samples were shaken for 12 hours at 160 RPM at room temperature (Fig. 1 D). The resultant cellulose scaffolds were then transferred into new sterile microcentrifuge tubes, washed and incubated for 6 hours in PBS (Sigma-Aldrich) with 1% streptomycin/penicillin (HyClone) and 1% amphotericin B (Wisent). At this point, the samples were immediately used or stored in PBS at 4°C for no more than 2 week.

### Post-decellularization treatments

Here, we examined cell proliferation and invasion into native, collagen functionalized, or chemically cross-linked cellulose scaffolds. In order to functionalize scaffolds with collagen, samples were incubated for 6 hours in a solution of 10% acetic acid and 1 µg/mL rat tail collagen type I (Invitrogen), followed by washing in PBS before use. To chemically cross-link the scaffolds, the samples were incubated in a 1% EM-grade glutaraldehyde solution (Sigma-Aldrich) for 6 hours. The scaffolds were then rinsed in PBS and incubated in a solution of 1% sodium borohydride (Sigma-Aldrich) overnight in order to reduce any unreacted glutaraldehyde (Fig. 1E). Prior to seeding cells into the scaffolds, all samples (native, collagen coated, or cross-linked) were incubated in mammalian cell culture medium (described below) for 12 hours in a standard tissue culture incubator maintained at 37°C with 5% CO_2_ (Fig. 1F).

### Cell culture

C2C12 mouse myoblasts, NIH3T3 mouse fibroblasts and HeLa human epithelial cell lines were used in this study (all obtained from the American Type Culture Collection (ATCC)). Cells were cultured in standard cell culture media (high glucose DMEM (HyClone), supplemented with 10% fetal bovine serum (HyClone), 1% penicillin/streptomycin (HyClone) and 1% amphotericin B (Wisent) at 37°C and 5% CO_2_ in T75 flasks (Thermo Scientific). Culture media was exchanged every second day and the cells were passaged at 80% confluence.

### *In vitro* cell culture in cellulose scaffolds

The scaffold seeding procedure took place in 24-well tissue culture plates. Each well was individually coated with polydimethylisiloxane (PDMS) to create a hydrophobic surface in order to prevent the adhesion of cells. A 1:10 solution of curing agent: elastomer (Sylgard 184, Ellsworth Adhesives) was poured into each well. The PDMS was cured for 2 hours at 80°C, and was allowed to cool to room temperature, then rinsed with PBS. Scaffolds were cut into 0.5×0.5 cm pieces and placed within each well. A 40 µL droplet containing 6×10^6^ cells was carefully formed on top of each scaffold. The samples were placed in the incubator for 6 hours to allow the cells to adhere to the scaffolds. Subsequently, 2 mL of DMEM was added to each well and the samples were incubated for 48 hours. At this point, samples containing mammalian cells were then carefully transferred into new 24-well PDMS-coated tissue culture plates. For continued cell proliferation, the culture media was exchanged every day and scaffolds were moved into new 24-well plates every 2 weeks.

### Immunofluorescence staining

The actin cytoskeleton and nucleus of mammalian cells, cultured on glass or within the scaffolds, were stained according to previous protocols [46], [47]. Briefly, samples were fixed with 3.5% paraformaldehyde and permeabilized with Triton X-100 at 37°C. Actin was stained with phalloidin conjugated to Alexa Fluor 488 (Invitrogen) and nuclei were stained by labelling the DNA with DAPI (Invitrogen). Samples were then mounted in Vectashield (Vector Labs).

In order to simultaneously stain the cellulose scaffold and mammalian cells, we first fixed the samples as described above, and then washed them with PBS 3 times. To label the apple cell walls, we used an established protocol described previously by Trueunit et al. (2008) [48]. The samples were rinsed with water and incubated in 1% periodic acid (Sigma-Aldrich) at room temperature for 40 minutes. The tissue was rinsed again with water and incubated in Schiff reagent (100 mM sodium metabisulphite and 0.15 N HCl) with 100 mg/mL propidium iodide (Invitrogen) for 2 hours. The samples were then washed with PBS. To visualize the mammalian cells within the apple tissue, the samples were incubated with a solution of 5 µg/mL wheat germ agglutinin (WGA) 488 (Invitrogen) and 1 µg/mL Hoechst 33342 (Invitrogen) in HBSS (20 mM HEPES at pH 7.4; 120 mM NaCl; 5.3 mM KCl; 0.8 mM MgSO_4_; 1.8 mM CaCl_2_; and 11.1 mM dextrose). WGA and Hoechst 33342 are live cell dyes that label the mammalian cell membrane and nucleus, respectively. The samples were then transferred onto microscope slides and mounted in a chloral hydrate solution (4 g chloral hydrate, 1 mL glycerol, and 2 mL water). Slides were kept overnight at room temperature in a closed environment to prevent dehydration. The samples were then placed in PBS until ready for imaging.

We also labelled samples to test for long-term mammalian cell viability. In these cases, cells were maintained in culture for 12 weeks and then stained with a solution of 1 µg/mL Hoechst 33342, which stains the nuclei of all cells, and 1µg/mL Propidium iodide (PI), which is cell membrane impermeable and will only stain the nucleic acids of apoptotic or necrotic cells. Samples were then fixed with 3.5% paraformaldehyde as above and then submerged in PBS until ready for confocal imaging. In order to quantify the number of viable cells we prepared and stained n = 3 samples. Individual Hoechst-positive and PI-positive cells were automatically counted using the particle analyzer function on ImageJ.

### Optical Microscopy

Confocal imaging was performed on an A1R high speed laser scanning confocal system on a TiE inverted optical microscope platform (Nikon, Canada) with appropriate laser lines and filter sets. Transmitted light images were acquired on an inverted TiE microscope (Nikon, Canada) with phase contrast optics. Images were analyzed using ImageJ open access software (http://rsbweb.nih.gov/ij/). Brightness and contrast adjustments were the only manipulations performed to images.

### Scanning Electron Microscopy

Scaffolds containing mammalian cells were first fixed with 3.5% paraformaldehyde as presented above, and then gently washed repeatedly with PBS. The samples were then dehydrated through successive gradients of ethanol (50%, 70%, 95% and 100%) and dried within a lyophilizer. Samples were then gold-coated at a current of 15 mA for 3 minutes with a Hitachi E-1010 ion sputter device. SEM imaging was conducted at voltages ranging from 2.00–10.0 kV on a JEOL JSM-7500F FESEM.

### Atomic force microscopy (AFM)

AFM was employed to measure the mechanical properties of the native, collagen functionalized, or chemically cross-linked (n = 3 in each case) cellulose scaffolds. In all cases, tip-less PNP-TR-TL (Nano world) AFM cantilevers were modified with a 10 µm polystyrene bead (Fluka) using an optical adhesive (Norland), cured in a UV cross-linker (Spectroline Select Series). Cantilevers possessed an average spring constant of 37±5 pN/nm as determined using the thermal fluctuation method [49], [50]. Local mechanical properties were measured by 5–15 force-indentation curves collected at 10–15 randomly chosen locations at a rate of 1 Hz. A total of n = 200 measurements were acquired for each sample. PUNIAS software was used to fit the first 200 nm of indentation to the Hertz model for a spherical indenter, using a Poisson ratio of 0.5 [46], [50], [51].

### Statistical Analyses

All presented values are the average ± standard deviation. Where applicable, we assessed statistical significance by performing a two-sample student's t-test (α<0.05).

## Results

### Preparation of cellulose scaffolds

**Figure 2:**
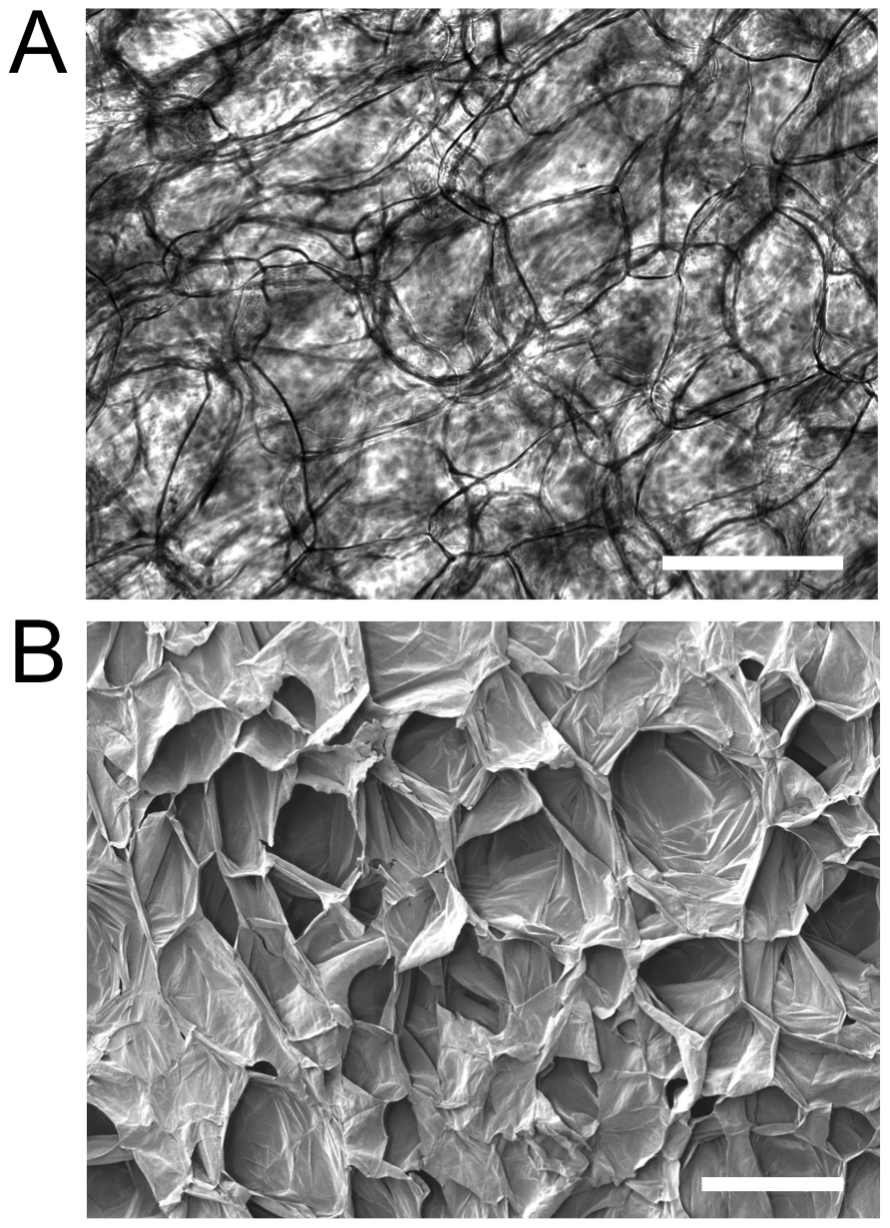
Decellularized cellulose scaffolds. A) Phase contrast image of cellulose cell wall structure in a decellularized apple tissue sample. The dark lines correspond to distinct cellulose structures which form a three dimensional matrix. B) SEM image of a similar cellulose scaffold revealing its three dimensional nature and large cavities. Scale bar = 200 µm.

As described in the Materials and Methods section, apple hypanthium tissue was cut to uniform size and decellularized following established protocols (Fig. 1) [41]. Hypanthium tissue was employed as it is rich in cellulose and contains very few cells [52], [53] Decellularization protocols were employed to ensure the complete removal of any remaining plant cells, nucleic acids and biomacromolecules. After processing the samples, a highly porous structure is observed with phase contrast microscopy (Fig. 2A). The apple tissue has evolved as a very porous structure, with cell wall cavities observed throughout the sample, allowing for facilitated nutrient transfer throughout. Cellulose scaffolds were then fixed and dehydrated for SEM imaging. Samples were cut horizontally down the mid-section revealing the interior surface. A highly porous and relatively robust scaffold is clearly observed (Fig. 2B). In all cases, the cellulose scaffold was the only apparent feature observed in all images, as no other identifiable structures were witnessed (i.e. cellular organelles or otherwise).

### Mechanical properties of native and modified cellulose scaffolds

**Figure 3:**
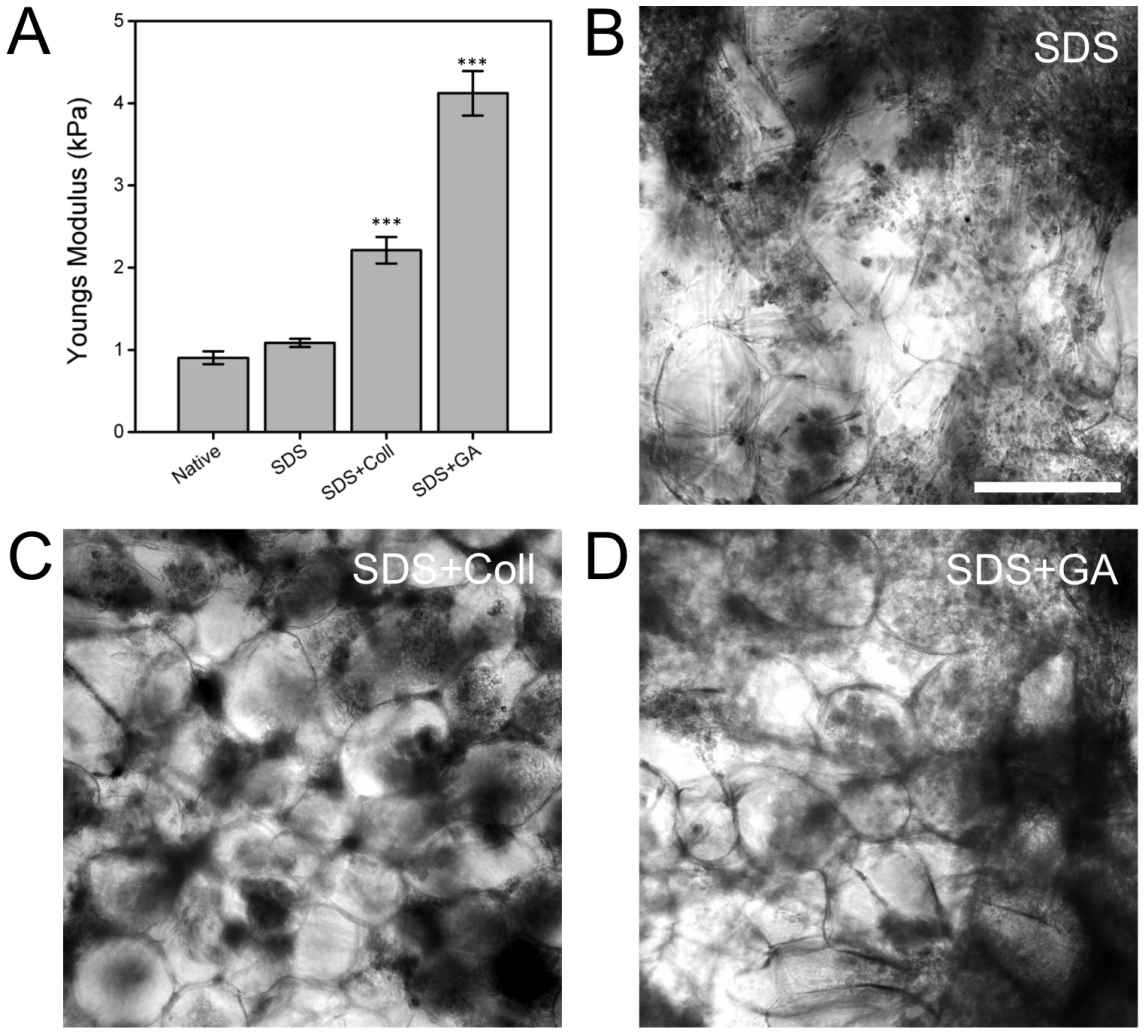
The mechanical properties of functionalized cellulose 3D scaffolds and C2C12 myoblast cultured within the 3D cellulose scaffolds. A) The local mechanical elasticity of native tissue, decellularized (SDS), collagen functionalized (SDS+Coll) and glutaraldehyde (SDS+GA) cross-linked cellulose scaffolds. The native tissue and unmodified scaffolds do not display any significant difference in mechanical properties. Both the collagen functionalized and chemically cross-linked scaffolds displayed a significant increase in elasticity compared to the DMEM scaffolds (*** = *p*<0.001). The (B) decellularized (SDS), (C) Collagen functionalized (SDS+Coll) and (D) glutaraldheyde cross-linked (SDS+GA) scaffolds all support the growth of C2C12 cells. Phase contrast images of C2C12 cells after two weeks of growth reveal the presence of cell colonies. Scale bar = 200 µm.

We employed two post-decellularization functionalization protocols in order to examine the ease of modification of the mechanical properties of these cellulose scaffolds. The two modifications included functionalization of the scaffold with type I collagen, or chemically cross-linking of the scaffold with glutaraldehyde. These modifications allowed us to control the biochemical environment of the scaffolds, and alter their mechanical properties. AFM was used to quantify the local elasticity of the scaffolds in response to each treatment. We measured the elasticity of four specific samples: untreated (native), decellularized (SDS), decellularized and collagen functionalized (SDS+Coll), and finally decellularized and glutaraldehyde cross-linked (SDS+GA) tissue. Native tissue, SDS, SDS+Coll and SDS+GA scaffolds possessed an elasticity of 0.9±0.1 kPa, 1.1±0.1 kPa, 2.2±0.2 kPa and 4.1±0.3 kPa, respectively (Fig. 3A). The native and SDS scaffolds did not display any significant difference in mechanical properties (*p*>0.05). Both the SDS+Coll and SDS+GA scaffolds displayed a significant increase in elasticity compared to the native and decellularized scaffolds (*p*<0.001). These results demonstrate that the local elasticity of the scaffolds can clearly be controlled to fall within a range which mimics some mammalian tissues [54], [55] Finally, C2C12 cells were seeded into different scaffold preparations (SDS, SDS+Coll and SDS+GA, n = 3 in each case) and cultured for 2 weeks (Fig 3B-D). Phase contrast microscopy revealed the presence of mammalian cells in each of the scaffolds compared to the cellulose scaffold presented in Fig. 2A. The images shown are representative of the n = 3 scaffolds prepared in each case.

### Mammalian cell culture in native, collagen functionalized, and chemically cross-linked cellulose scaffolds

**Figure 4:**
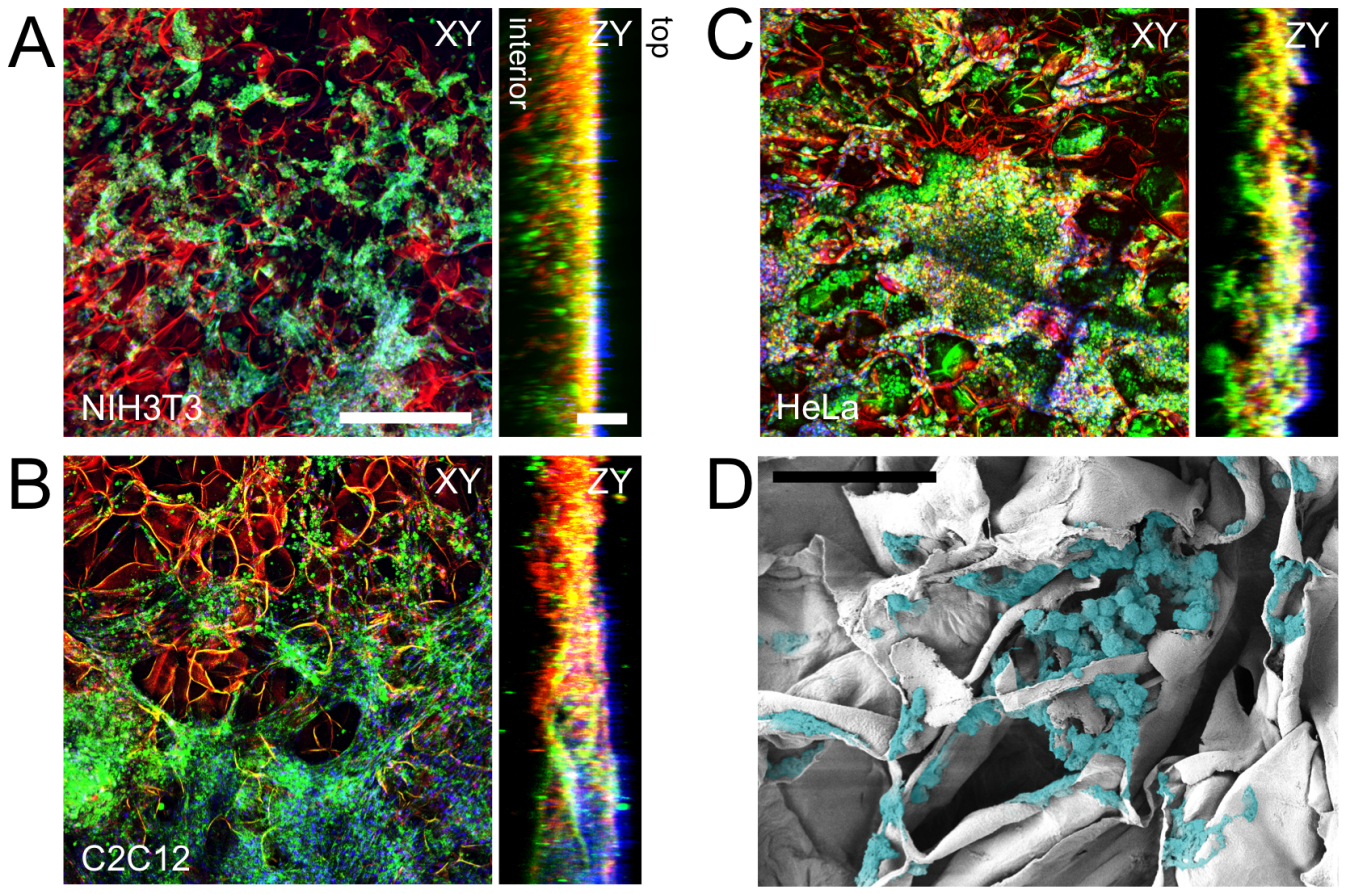
Fixed and stained NIH3T3, C2C12 and HeLa cells cultured on 3D cellulose scaffolds. Specific fluorescent staining of (A) NIH3T3, (B) C2C12 and (C) HeLa mammalian cells within the cellulose scaffolds and subsequent laser scanning confocal microscopy reveals the cellulose structure (red), mammalian cell membranes (green) and nuclei (blue). Cells were cultured in these scaffolds for four weeks prior to staining and imaging. Confocal volumes were acquired and projected in the XY and ZY plane. The ZY orthogonal views demonstrate the depth of cell proliferation within the cellulose scaffold. The top and bottom surfaces of the scaffold are indicated. Scale bars: XY = 300 µm, ZY = 100 µm. D) SEM image of a cellulose scaffold cross section after being seeded with C2C12 cells that were allowed to proliferate for four weeks. The cells were digitally colourized in order to increase contrast between the cells and cellulose structure (Scale bar: 50 µm)

To test the ability of the cellulose scaffolds for supporting 3D mammalian cell culture, we examined the proliferation of mouse C2C12 myoblasts, mouse NIH3T3 fibroblasts and human HeLa epithelial cells within the native, collagen functionalized, or chemically cross-linked cellulose scaffolds (n = 3 for each case). In all cases, proliferation of cells was similar. After 4 weeks in culture, immunofluorescent images were generated for every sample, to selectively visualize the mammalian cells within the apple cell wall. A distinct cell wall structure was observed for all conditions, seen as a uniform thin red structure forming individual cavities of consistent size (see Fig. 4). Interestingly, similar observations were made of the cellulose structure in the SEM images (Fig. 2b). The cell membranes (green) and nucleus (blue) of C2C12 myoblasts (Fig. 4A), mouse NIH3T3 fibroblasts (Fig. 4B) and human HeLa epithelial cells (Fig. 4C) clearly proliferate very well in unmodified (Fig. 4A-C), as well as modified scaffolds (data not shown). Cells were observed to be growing on the surface of the scaffolds.

It was also observed that the majority of cells are in closely interacting aggregates within the individual cell wall cavities. To further examine the proliferation within the cellulose structure, orthogonal views of volume-images were generated. The green (membrane) and blue (nucleus) signals can be seen within the interior of the cellulose structure, thus demonstrating that the cells proliferate deep within the scaffolds. Fluorescent signals can be seen up to depths of approximately 120 µm from the surface of the scaffolds. It is important to note that the loss of fluorescence signal coincides with the limit of imaging depth of the confocal microscope. Hence, the actual degree of cell invasion into the scaffold cannot be determined with confocal microscopy alone. To further investigate the degree of cellular invasion into the cellulose scaffolds we fixed and dehydrated (n = 3) native scaffolds for SEM imaging. The samples were prepared in a way that revealed the interior surface of the cellulose scaffolds. SEM images also reveal that cells can migrate within the scaffolds. In Fig 4D, we present images of C2C12 cells that have migrated towards the interior of the sample. Cells are clearly visible within the scaffold (Fig. 4D) in comparison to SEM of an un-seeded scaffold (Fig. 2B). Cells in the scaffold can be seen attached to the cellulose structure, with their morphologies varying between round and spread, consistent with other mammalian cells grown in other natural and synthetic 3D scaffolds [56]–[58].

**Figure 5:**
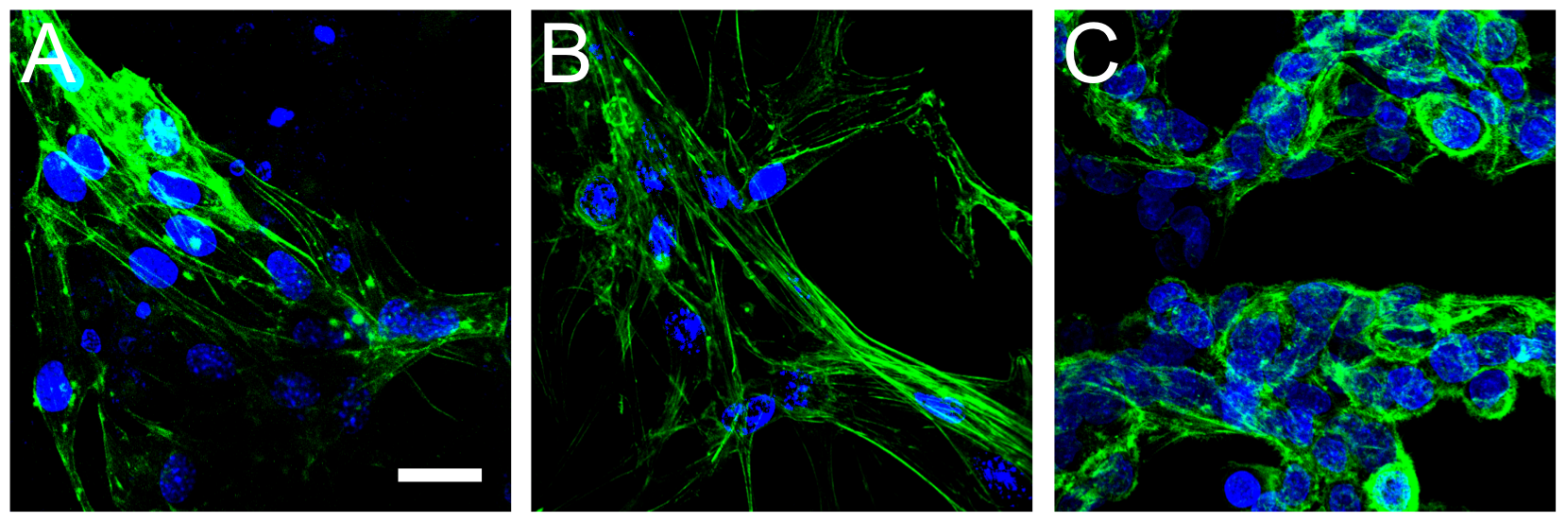
Fixed and stained images of cells actin cytoskeleton cultured within the 3D cellulose scaffold. A) NIH3T3, B) C2C12 and C) HeLa cells were cultured onto the cellulose scaffolds for 2 weeks prior to stained for actin (green) and cell nuclei (blue). (. NIH3T3 and C2C12 cells display characteristic actin stress fibres found in cultured cells. HeLa cells also display characteristic actin structures including fewer prominent stress fibres and a large amount of cortical actin localization. Scale bar = 25 µm and applies to all.

Finally, the morphology of the actin cytoskeleton was also examined for all cell types in unmodified cellulose scaffolds (n = 3 for each cell type). In this case, cells were only cultured for 1 week, as the actin cytoskeleton was more easily resolved in lower density samples. All mammalian cell types within the scaffolds displayed clear actin stress fibers, consistent with strongly attached cells, as those demonstrated on solid 2D substrates [46], [47], [59] (Fig. 5). These results demonstrate that the mammalian cells were well-adhered to the cellulose scaffold, rather than simply being physically confined within the scaffold.

### Proliferation and Viability

**Figure 6:**
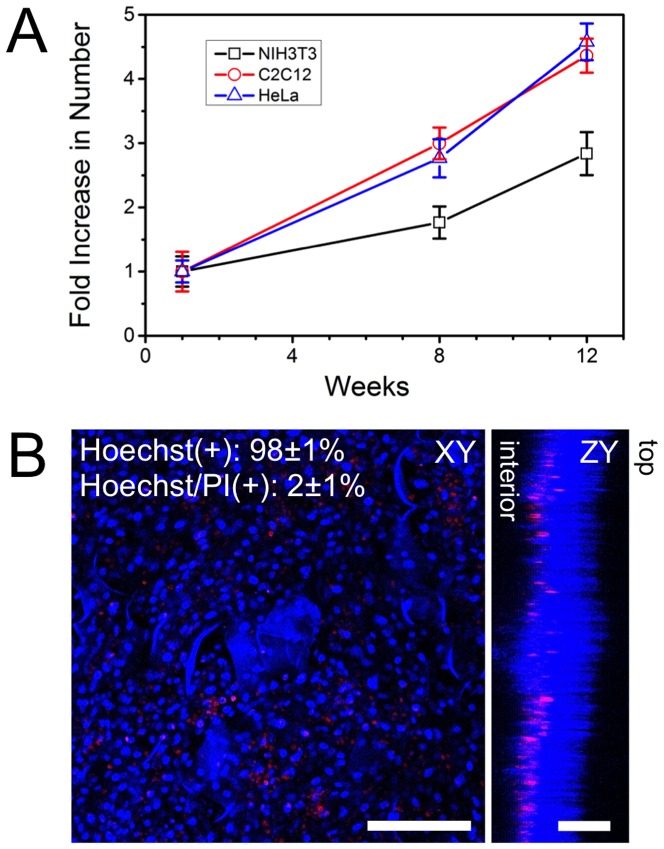
Cell proliferation and viability over time. A) NIH3T3, C2C12 and HeLa cells were cultured individually in cellulose n = 3 scaffolds for 1, 8 and 2 weeks and then imaged with confocal microscopy after being stained with Hoechst 33342. Cells were counted at each time point and an increase in cell population is clearly observed. B) After 12 weeks of culture, C2C12 cells were fixed and stained with Hoechst 33342 (blue: viable cells) and Propidium iodide (PI) (red: apoptotic/necrotic cells). Confocal volumes were acquired and projected in the XY and ZY plane and reveal that cells have proliferated throughout the structure during the 12-week culture. The cells that are apoptotic/necrotic are found in deeper regions of the scaffold. The top and bottom surfaces of the scaffold are indicated. The number of live (Hoechst(+)) and dead (Hoechst/PI(+)) cells were counted and it was found that ∼98% of the cells within the scaffold are viable. Data is shown for C2C12 cells, but is similar for NIH3T3 and HeLa cells (data not shown). Scale bar: B = 200 µm for XY and 100 µm for ZY.

In order to quantitatively assess long-term cell proliferation and viability, confocal images were acquired throughout the cellulose scaffold after 1, 8 and 12 weeks of continuous cell culture. To assess proliferation, confocal volumes of cell nuclei were acquired at random locations on n = 3 scaffolds seeded with NIH3T3, C2C12 or HeLa cells. For each cell type, n = 3 randomly chosen 1.6×10^6^ µm^2^ imaging regions were chosen, and confocal imaging was performed. The imaging depth required to capture all cells in the sample area was observed to change due to the fact that cells were invading deeper into the scaffold over time. Using this data, cells were counted and shown to increase in number by several-fold over a 12-week period (Fig. 6A). HeLa and C2C12 cells were observed to increase in number at a similar rate, which was higher than NIH3T3 cells. Regardless, all cell types exhibited a three- to four-fold increase in number over a 12-week period of continuous culture.

We also examined cell viability within these cellulose scaffolds after 12-weeks of continuous culture. Scaffolds (n = 3) were incubated with Propidium iodide and Hoechst 33342 to specifically label apoptotic/necrotic or living cells, respectively (Fig. 6B). In all cases, Hoechst labelled all cell nuclei (live and dead) throughout the sample. Conversely, Propidium iodide specifically labelled a minority of dead cells, which were mainly located towards the interior portion of the scaffold. Cell viability was quantified by counting the Hoescht-positive (live) and PI/Hoescht-positive (dead) nuclei of cells. The majority of cells (98±1% of C2C12 cells) cultured on the apple scaffold were Hoechst-positive after 12 weeks in culture (Figure 6C), with identical results in the other cell types (data not shown). These results demonstrated a high degree of viability for long-term cultures on the surfaces of these cellulose scaffolds. Orthogonal projections demonstrated that a small population of apoptotic/necrotic cells were located approximately 120 µm towards the interior of the cellulose scaffold (see Fig. 6B). Even in regions of increased cell density there was sufficient porosity within the cellulose scaffold to allow for media/nutrient transfer to the interior of the scaffolds.

## Discussion

Cells in a 3D environment, whether an artificial extracellular matrix or living tissues, often display numerous morphological and biochemical differences compared to cells cultured on a 2D surface [8]. These differences are under intense scrutiny, as many studies have demonstrated the importance of spatial cues within the ECM. For example, geometry and special cues have been shown to affect cell morphology, mRNA signalling, and differentiation [5], [9], [60]–[62]. Therefore, the use of 3D scaffolds for in vitro cell culture is beneficial towards our fundamental understanding of cell biology, and has consequently has many potential applications. The development and use of 3D biocompatible scaffolds does however present several challenges. Biomaterials must be biocompatible, biodegradable, allow for surface modifications, and be cost effective. Two methods of production have emerged. The first of these methods, is the use of artificial scaffolds synthesized from (bio)polymers. Artificial scaffolds have the advantage of allowing for exceptional control over the material, allowing for tuning of biochemical and structural properties of the scaffold [63]–[67]. On the other hand, decellularization has been employed to produce natural 3D scaffolds from existing tissue [13], [33], [44], [65]–[68]. Decellularization employs various reagents to lyse and remove cells from the ECM of a given tissue sample [32], [34]. Though this approach lacks fine control over the physical and biochemical properties of the scaffold, it results in an easily obtained, naturally derived, scaffold that has been employed in the creation of functional organs [33], [41]–[44].

Herein, decellularized apple hypanthium tissue is shown to provide several characteristics that are desirable for producing 3D cellular scaffolds. Apple hypanthium is primarily composed of plant cell walls which form a porous network of cavities in which the plant cells reside [69]. This porous environment allows for facilitated transfer of nutrients throughout the tissue. In addition, this environment is also ideal for decellularization as it allows for rapid exchange of detergents, buffers and media without the use of perfusion systems. We were able to rapidly produce decellularized cellulose scaffolds by simply using a detergent solution. Importantly, mouse fibroblast (NIH3T3), mouse muscle myoblast (C2C12) and human epithelial (HeLa) cells were all able to completely invade, proliferate and remain viable after a long term period of culture (12 weeks) within the cellulose scaffolds.

The mechanical and biochemical properties of the ECM are known to play a very important role in governing cellular physiology [5], [60]–[62]. The apple hypanthium-derived scaffolds, used in this study, are composed of cellulose that is assembled into microfibrils which are then cross-linked by hemicellulose [70], and resemble the porous nature of the ECM. Here, using AFM, we demonstrated that the local Young's modulus of the scaffold could be altered through chemical cross-linking or biochemical functionalization. We found that the native and SDS samples displayed no statistical difference in local Young's modulus (*p*<0.05), demonstrating that SDS treatment did not alter the mechanics of the cellulose structure [31]. Furthermore, it was also observed that scaffolds functionalized with collagen or cross-linked with glutaraldehyde displayed a significant two to four-fold increase in Young's modulus (*p*<0.001) compared to the native tissue or unfunctionalized decellularized scaffolds. Our results are consistent with previous studies, which demonstrate that collagen functionalization or glutaraldehyde cross-linking tends to increase the Young's modulus of biological materials [71]–[73]. The ability to tune the cellulose scaffold rigidity is critical as it has been long established that cells preferred matrix with specific rigidities to differentiate and acquire their specific cellular properties [32], [34], [60], [69], [70]. Hence, we showed that it is possible to control and modulate the mechanical rigidity of the cellulose scaffold.

Previous studies employing mechanical compression testing of bulk apple samples have reported significantly higher Young's modulus measurements, in the MPa range [74], [75], compared to the ones obtained in our study. Their technical approaches measure an elasticity that reflects the forces required to deform all of the cells and cellulose structures within a large region of apple tissue. In contrast, we employed an AFM with a modified cantilever probe using a 10 µm colloid polystyrene bead in order to probe local elasticities. Young's modulus measurements obtained with this approach are reflective of the local deformation of a small microscale region of the cellulose scaffold. Due to the local nature of AFM measurements, the measured Young's modulus more accurately reflects the mechanical properties experienced by the cells. Interestingly, in a recent study, the local Young's modulus of tomato plant tissue was determined to be in the same order of magnitude of our measurements, ∼7–29 kPa [76]. These results were also obtained using AFM and a modified cantilever with an attached 10 µm colloid. Importantly however, under all conditions and for differing Young's moduli, cells were observed to rapidly grow and proliferate within the cellulose scaffolds.

Employing a staining protocol established by Truernit *et al.* [48], we simultaneously visualized the cellulose structure along with the embedded cells. Confocal microscopy images revealed that all three cell types used in this study were found on the surfaces and the interior cavities within the cellulose scaffold. Cells were reliably imaged to a depth of approximately 120 µm below the upper surface of the scaffold. In all three cell types we also observed the presence of distinct F-actin stress fibres, which are a morphological characteristic of substrate-adhered cells [47], [77], [78]. Both fibroblasts and myoblasts cultured in our scaffolds possessed pronounced actin stress fibres, similar to those found in the same cells cultured on 2D substrates [46], [47]. HeLa cells were observed to express cortical actin and actin stress fibres. In this case, stress fibres were less prominent than those observed within the fibroblasts and myoblasts, however the observed morphology of the actin cytoskeleton is very consistent with HeLa cells cultured on 2D substrates [78]. The results clearly demonstrate that all cell types studied here were able to proliferate, displaying strong attachment to the surfaces of the cellulose scaffolds.

A constraint of confocal microscopy is the limited depth at which a sample can be imaged. In order to determine whether the mammalian cells migrated towards the interior of the scaffold, the sample was fixed, frozen, and then fractured perpendicular to the top surface. This process allowed us to image the interior of the cellulose scaffold using SEM. In this case, C2C12 cells were cultured on the scaffolds and subsequently imaged. Cells were found deep within the interior of the scaffold and to possess morphologies that were both rounded, as well as flat and spread-out along the cellulose surfaces, analogous to that of myoblasts cells in other 3D environments [79], [80]. Interestingly, the cellulose scaffold appears in a collapsed/compressed state in the presence of mammalian cells. When mammalian cells are absent from the scaffold, such morphologies are not observed, confirming that the preparation for SEM imaging is not causing this effect. At present, the origin of this change in morphology is not well understood, however we hypothesize it may be due to the presence of cellular traction forces, adsorption of serum proteins on the scaffold, or cellular remodelling/degradation of the scaffold.

To quantify proliferation, we counted the number of cells present in the scaffolds after 1,8 and 12 weeks of continuous *in vitro* culture. In all cases, cells were observed to proliferate and increase in number by three to four-fold. However, it was observed that C2C12 and HeLa cells had nearly twice the number of NIH3T3 cells. This is an intriguing result as doubling rate of NIH3T3 cultured on glass (18 hours) is slightly lower than that of HeLa cells (24 hours) [81], [82]. Viability assays also reveal that a high percentage of cells remained viable even after 12 weeks of continuous in vitro culture. The majority of apoptotic cells were present towards the interior of the cellulose scaffold. This is likely caused by a lack of media turnover, leading to an insufficient supply of nutrients and oxygen, a common phenomenon in 3D culture models [83]. 3D spheroid cell cultures that often contain a necrotic core of cells due to an insufficient supply of nutrients and oxygen [83].

Although the results show that cells proliferate and remain viable in the scaffolds, their porosity can have an important influence on the observed results. The high porosity of the scaffolds offers both advantages and disadvantages. The high porosity of the scaffold allows cells to pass through the material without a high degree of confinement. This leads to a low seeding efficiency that can be problematic in some applications (the culture of rare cells, etc.). For example, in this study each scaffold (∼1 mm^3^ total volume) was initially seeded at a density of ∼6×10^6^ cells/mm^3^. However, cell density was lower after 1-week of culture (∼2×10^6^ cells/mm^3^) due to the loss of cells during media exchange. Therefore, a large amount of time (12-weeks) is required for the cells to slowly proliferate and invade the entire scaffold. This is not surprising given the issues highlighted above, and the fact that these mammalian cells are proliferating in a scaffold that is composed largely of cellulose as opposed to ECM proteins. Although there is a slow proliferation, the cell density does approach the levels reported in other biomaterials [41], [43], [84]. The efficiency of the initial seeding might be improved through further biochemical modification or chemical cross-linking of the scaffolds to increase the binding potential. Future work will investigate scaffold modification to control porosity and improve seeding efficiency. Alternatively, naturally occurring cellulose with lower porosity might be found in other apple species or plant types. Regardless, future studies will examine the use of higher density cellulose scaffolds to increase seeding efficiency, which will lower the time required to fully populate the scaffolds.

In addition, the high porosity of the scaffolds likely leads to an overestimation of cell viability. Dead cells can be easily washed away during media exchange, lowering the observed number of dead cells in the scaffold. Furthermore, the limited depth associated with confocal microscopy will also influence the ability to assay the physiological state of the cells deep within the scaffold. Other viability assays are also impeded by the nature of the cellulose scaffold. In particular, Trypan blue assay is problematic as the dye interacts strongly with the scaffold, which significantly impedes cell counting and quantification.

Although these limitations exist, mammalian cells are clearly able to proliferate, invade and possess an intact actin cytoskeleton within the scaffolds. In all three cases, cells were observed to interact closely with each other and the scaffold in large, long-range networks. Furthermore, theses cellulose scaffolds represent a very low-cost approach to studying the proliferation and invasion of cells in 3D. These cellulose biomaterials are highly complementary with the myriad of possibilities for the study of 3D cell biology *in vitro*. Cellulose scaffolds are porous enough to allow for efficient nutrient/gas exchange, biocompatible, easily functionalized and their mechanical properties can be controlled.

## Conclusions

The results presented in this study naturally give rise to questions about the *in vivo* applicability of cellulose scaffolds. At this point, it is still too early to speculate, as there are many unknowns about the biocompatibility of these scaffolds *in vivo*. The immunological response and the long-term stability of implanted cellulose-based biomaterials are still being studied [22], [85]. We have not examined these issues in this study, as our objective was only to demonstrate the suitability of apple-derived cellulose scaffolds in supporting the *in vitro* culture of mammalian cells. Numerous approaches for producing 3D matrices that support the culture of mammalian cells are available [86], however, many of these products are proprietary, expensive, or require chemical synthesis. Plant derived cellulose scaffolds offer an alternative approach for 3D culture, offering the advantage of ease of production and modification, reduced cost, the ability to fabricate the cellulose into shapes specific to the user. Taken together, our results demonstrate that natural apple-derived cellulose scaffolds can be produced by employing common decellularization approaches and will support 3D cell culture. The evolved porosity of the apple hypanthium tissue facilitates the initial decellularization and provides critical media transfer allowing for long-term cell viability. Three mammalian cell types were observed to proliferate, migrate, and remain viable in the scaffolds for up to 12 weeks (maximum length of study). In addition, biochemical functionalization or chemical cross-linking can also be employed to control the surface biochemistry and/or mechanical properties of the scaffold. The advantages of these cellulose scaffolds make them one of several potential biomaterial candidates for in vitro 3D cell culture that are currently available.
